# The Psychological Impact of Prenatal Diagnosis and Disclosure of Susceptibility Loci: First Impressions of Parents’ Experiences

**DOI:** 10.1007/s10897-016-9960-y

**Published:** 2016-05-25

**Authors:** S. L. van der Steen, S. R. Riedijk, J. Verhagen-Visser, L. C. P. Govaerts, M. I. Srebniak, D. Van Opstal, M. Joosten, M. F. C. M. Knapen, A. Tibben, K. E. M. Diderich, R. J. H. Galjaard

**Affiliations:** 1 Department of Clinical Genetics, Erasmus Medical Centre, Rotterdam, The Netherlands; 2 Department of Obstetrics and Prenatal Medicine, Erasmus Medical Centre, Rotterdam, The Netherlands; 3 Stichting Prenatale Screening Zuidwest Nederland, Rotterdam, The Netherlands; 4 Department of Clinical Genetics, Leiden University Medical Centre, Leiden, The Netherlands

**Keywords:** Invasive prenatal diagnosis, Susceptibility loci, VOUS, CNV, Psychological impact, Genomic microarray, Uncertainty, Individualized choice

## Abstract

Genomic microarray may detect susceptibility loci (SL) for neurodevelopmental disorders such as autism and epilepsy, with a yet unquantifiable risk for the fetus. The prenatal disclosure of susceptibility loci is a topic of much debate. Many health care professionals fear that reporting susceptibility loci may put a psychological burden on pregnant couples. It is our policy to disclose prenatal susceptibility loci as we recognize them as actionable for prospective parents. The aim of this report was to evaluate the psychological impact of disclosing a prenatal diagnosis of susceptibility loci. The psychological impact of disclosing susceptibility loci was evaluated in the first patients who received such results. Eight out of 15 women who had a susceptibility locus disclosed and four of their partners consented to share their experiences through a telephonic evaluation (*n* = 12). Follow-up time ranged from 3 to 15 months after their prenatal test result. The reporting of susceptibility loci was initially ‘shocking’ for five parents while the other seven felt ‘worried’. Ten out of 12 participants indicated they would like to be informed about the susceptibility locus again, two were unsure. Most had no enduring worries. Participants unanimously indicated that pregnant couples should have an individualized pre-test choice about susceptibility loci (non)disclosure. We observed no negative psychological impact with the prenatal diagnosis and disclosure of SL on participants. A key factor in mitigating parental anxiety with SL disclosure appears to be post-test genetic counseling. Our report confirms that pregnant women and their partners prefer an individualized choice regarding the scope of prenatal testing.

## Introduction

Genomic microarray may detect more copy number variants (CNVs) that cause clinically relevant abnormalities and generates results faster than conventional karyotyping (CK) (Wapner and Jackson 2008; Wapner et al. 2012; Fiorentino et al. 2011). Therefore, we use SNP array instead of conventional karyotyping (CK) for routine cytogenetic analysis for all indications since July 2012 (Srebniak et al. 2013; Van Opstal et al. 2015). Next to known microdeletion syndromes such as Prader-Willi syndrome, or Duchenne muscular dystrophy, array testing may also reveal susceptibility loci (SL) for neurodevelopmental disorders. Susceptibility loci (SL) were defined as following by Girirajan et al.: ‘SL are copy number variants (CNVs) with an extreme phenotypic heterogeneity and/or of variable expressivity’ (Van Opstal et al. 2015; Girirajan et al. 2012; Srebniak et al. 2014, 2015) associated with an unquantifiable risk of neurodevelopmental disorders such as epilepsy, autism and psychiatric disorders and can be found in about 1.4 % of fetuses without ultrasound anomalies (Van Opstal et al. 2015; Srebniak et al. 2015). SL are often inherited from (apparently) unaffected parents, but are more frequently detected in affected individuals as compared to control populations (Srebniak et al. 2014; Kaminsky et al. 2011; Rosenfeld et al. 2013; Srebniak 2013). Genetic counseling in pregnancies where SL are found is challenging as it is difficult to estimate the chance of expression and/or to predict the phenotype because most likely a second hit like another genetic or even non-genetic factor, like environment, may also influence the expression of the phenotypes (Veltman and Brunner 2010; Girirajan et al. 2010). Almost all information about SL phenotypes and penetrance that is available is based on postnatal ascertainment. There is currently no information available about the development of children in whom a SL was found prenatally.

The value of SNP array in fetuses who were prenatally diagnosed with ultrasound anomalies has been widely accepted (Wapner et al. 2012; Fiorentino et al. 2011), but its implementation for other indications has raised concerns among health care professionals, causing much debate regarding the disclosure of SL (de Jong et al. 2013; McGillivray et al. 2012; Vetro et al. 2012). Some classify these CNVs as variants of unknown clinical significance (VOUS) (Wapner et al. 2012), but because of their association with an abnormal phenotype, we have classified SL as pathogenic (Srebniak et al. 2014). In our opinion, SL are different from VOUS because the phenotypic effect of VOUS is unknown, whereas for an SL the association with a specific phenotype is known but has a highly variable penetrance and expression.

It has been argued that pregnant couples may wish not to be informed on findings of uncertain expression (Hillman et al. 2013) and that such findings should be withheld in order not to put burden on the pregnant couple (Rigter et al. 2013; de Jong et al. 2014). It has also been said, both for susceptibility loci and VOUS, that reporting them may create a false sense of autonomy (Brady et al. 2013), because an overload of information could deteriorate reproductive autonomy, or raise possible emotional harm such as distress (McGillivray et al. 2012). Some ethicists argued that genetic information of unclear meaning interferes with reproductive autonomy and should not be provided for this reason (de Jong et al. 2014).

On the other hand, others argue that it is paternalistic to try to prevent women from emotional harm and potential termination of a pregnancy, and that pregnant women are entitled to be informed of all genetic information (McGillivray et al. 2012) and that better tools for dealing with uncertainty should be developed (Vetro et al. 2012; Rigter et al. 2013; Wolf et al. 2008; Stark et al. 2013).

Although we are well aware of the burden that SL may represent psychologically for the pregnant couple, for several reasons we have chosen to disclose SL when prenatally detected. Firstly, we consider most SL to be actionable during and/or after pregnancy. For example, SL may be associated with congenital heart disease and an expert ultrasound examination during pregnancy can be offered. Secondly, if neurodevelopmental problems occur (either early or late onset), rapid diagnostics and more adequate care may be mobilized when parents have the knowledge of the SL (Govaerts et al., manuscript in preparation) (Dababnah and Parish 2015).

Since we implemented SNP array for all indications, we encountered 14 cases of SL in 1330 pregnancies without ultrasound abnormalities (Van Opstal et al. 2015). To date, no patient experiences regarding the psychological impact of SL on pregnant couples has been reported. To explore whether disclosure of SL indeed puts a heavy burden on the parents (McGillivray et al. 2012; Rigter et al. 2013; de Jong et al. 2014; Brady et al. 2013), we feel it is important to understand how SL disclosure affects pregnant couples. We report on the narratives of 12 parents’ experiences with a prenatally disclosed SL.

## Patients and Methods

### Summary of the Standard Clinical Procedure

#### Pre-Test Counselling by a Senior Obstetrician

All patients undergoing invasive prenatal diagnosis (PND) received pre-test counselling by a senior obstetrician and received a patient information leaflet which specified that ‘all pathogenic results will be reported’. Pregnant couples were informed about array testing. The occasional occurrence of unexpected findings was discussed. These could either be pathogenic CNVs not related to the prior indication for invasive testing or susceptibility loci (SL), Patients received no detailed information regarding SL. Unexpected findings were discussed, but there was no strong emphasis on SL as a category of outcomes of invasive prenatal testing. SNP array testing was performed as a first-tier diagnostic test as described before (Srebniak et al. 2013; Van Opstal et al. 2015).

#### Disclosing the Prenatal Test Result

When a SL was diagnosed pregnant couples were contacted directly by a clinical geneticist informing them that there was no causative chromosomal abnormality found, but a deviant finding that may require special attention. They were invited for extensive post-test counseling available the next day. For extensive information about our counseling methods and pregnancy management, see Govaerts et al. (manuscript in preparation), in short:

1. The nature of the particular SL was explained. Phenotypic examples (including pictures) from the postnatal literature were available.
2. An expert ultrasound examination was offered if the SL was associated with structural abnormalities.
3. We offered targeted parental SNP array in all cases because knowing whether an SL was inherited aided in evaluating the clinical implications of the SL within the family.
4. The couples were informed about the possibility for early postnatal intervention programs (www.mee.nl), and the option to terminate the pregnancy was discussed.
5. The pregnant women and their partners were offered support from a medical psychologist specialized in prenatal care.

### Inclusion for the Psychological Evaluation

**Fig. 1:**
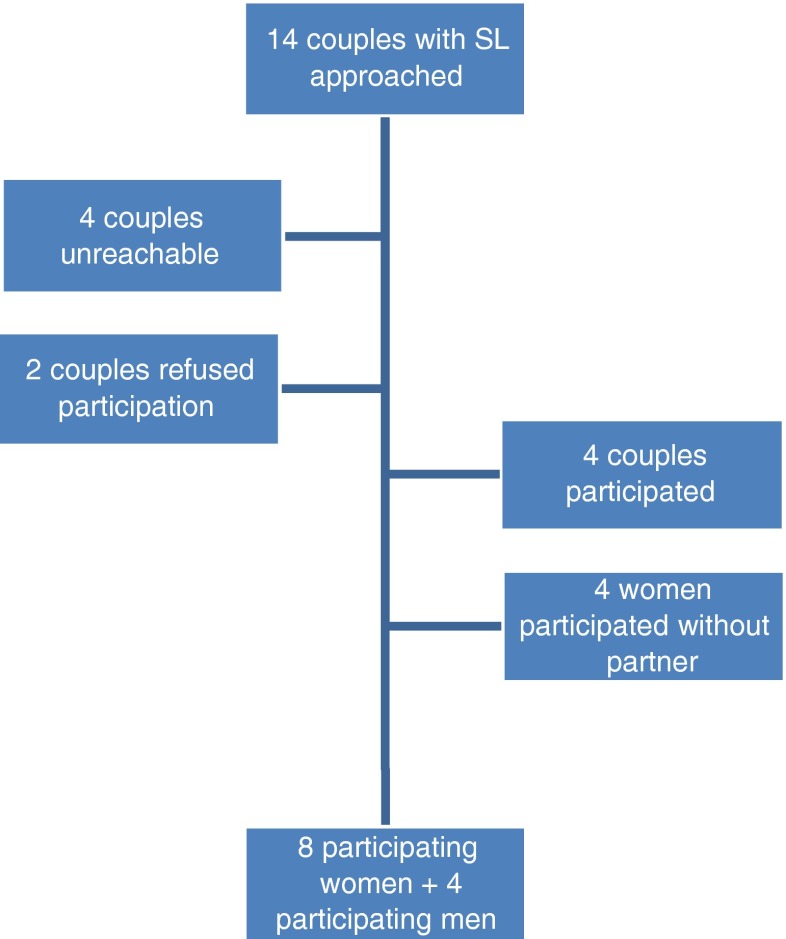
Participants

In this report we describe the experiences pregnant couples had when a susceptibility locus was found after invasive genetic testing, in the absence of ultrasound anomalies. Between July 2012 and December 2013, 14 couples received a prenatal diagnosis of a SL, and all of them were contacted. A clinical geneticist contacted them and asked whether they were willing to share their experiences by phone in order to assess the impact of disclosing a prenatal susceptibility locus. This interview was part of aftercare in order to learn about the long term psychological impact of SL disclosure in pregnancy. All patients proceeding with invasive prenatal testing, signed consent for further follow up during pregnancy and after delivery. Eight women and four of their partners agreed to share their experiences, see Fig. 1 for the participants. The prenatal testing indication and array findings in 8 fetuses of the parents that took part in the interview are shown in Table 1. None of the participants decided to terminate the pregnancy. The parents of live born children reported no congenital anomalies or dysmorphic features that were detected at birth. In Table 2, array results are displayed with phenotype and incidences based on the information the parents received. All couples were offered psychological support in dealing with the outcome after disclosure of SL, but none of them indicated they wanted to make use of this.

**Table 1:** Participants’ age, sex, prenatal testing indication, time of follow up interview after disclosure and array results with mode of inheritance; inherited from the mother (mat), de novo (dn), unknown in cases where parents refused testing (ukn)

Fetus	Participant	Age	Sex	Indication	Follow-up (months)	CNV (hg18) and inheritance
A	1	41	F	AMA	9	15q11.2 (20,191,584-20,710,960) x1 mat
B	2	41	F	aFTS	13	22q11.21 (17,249,767-19,959,004) x3 ukn
C	3	35	F	aFTS	8	16p11.2 (29,548,278-30,171,562) x3 dn
	4	35	M			
D	5	23	F	aFTS	15	15q11.2 (20,191,584-20,698,860) x1 ukn
	6	33	M			
E	7^a^	38	F	AMA	4	15q11.2 (20,191,584-20,698,860) x1 dn
	8	37	M			
F	9^a^	37	F	AMA	5	1q21.1 (144,959,767-146,307,651) x1 dn
G	10^a^	37	F	aFTS	3	15q11.2 (20,070,582-20,718,150) x1 mat
	11	38	M			
H	12	39	F	aFTS + AMA	15	3q29 (197,141,069-198,793,022) x3 mat

*AMA* advanced maternal age, *aFTS* abnormal first-trimester combined test

^a^Still pregnant during the interview

**Table 2:** Array results with incidences and postnatal ascertained phenotype

Array result	N	Incidences affected vs. controls^a^	Phenotype^b^
15q11.2 microdeletion	4	0.60 % vs. 0.20 % (Cooper et al. 2011) 0.41 % vs. 0.37 % (Burnside et al. 2011)	Intellectual disability, neurodevelopmental delay, behavioral problems, autism, facial dysmorphism (Girirajan et al. 2012; Rosenfeld et al. 2013)
22q11.2 microduplication	1	0.21 % vs. 0.05 % (Kaminsky et al. 2011)	Intellectual disability, hypotonia, hearing loss, epilepsy, cardiac malformations, urinary tract anomalies, growth retardation, facial dysmorphism (Firth 1993)
16p11.2 microduplication	1	0.18 % vs. 0.02 % [32] 0.25 % vs. 0.04 % (Kaminsky et al. 2011)	Intellectual disability, schizophrenia, autism (Kaminsky et al. 2011; Cooper et al. 2011)
3q29 microduplication	1	0.0005 % vs. 0.00009 % (Kaminsky et al. 2011)	Intellectual disability, hypotonia, occular anomalies, congenital heart defects (Ballif et al. 2008; Goobie et al. 2008)
1q21.1 microdeletion	1	0.35 % vs. 0.03 % (Kaminsky et al. 2011) 0.28 % vs. 0.018 % (Rosenfeld et al., 2013)	Intellectual disability, microcephaly, cardiac malformations, cataracts, schizophrenia, renal and urinary tract anomalies, autism (Rosenfeld et al. 2013)

^a^Incidences as counseled by the clinical geneticist in 2012–2013

^b^Phenotype: postnatal ascertained proband

The follow-up interview period between invasive PND and the contact ranged between 3 and 18 months. The mean time before the follow-up interview was 10 months after disclosure. Three participants were still pregnant at the time of follow-up.

### Measures

Consenting participants were approached for a follow-up interview by phone, using semi-structured questions (mean duration: 30 min). Women and their partners were interviewed individually. In Table 3, all questions are summarized. All interviews were transcribed verbatim and translated from Dutch to English. Worries about the health and development of the child were measured on a scale of 1 to 10 (1 - not at all to 10 - very much so).

**Table 3:** Open-ended questions about the psychological impact of prenatal SL disclosure

1. What was it like for you when you were told about the SL that was found?
2. What was your first reaction?
3. How do you feel about the SL at this very moment?
4. Would you choose to be informed of SL again?
5. Do you think that pregnant women should have a choice regarding the disclosure of SL?
6. Please indicate on a scale of 1 to 10 how worried you are about the health/development of your child? (1 not worried at all - 10 very worried)

### Analysis

Qualitative analysis was performed on the answers of all participants. A posteriori, three independent judges (SL; JV; SR) categorized the answers to the open-ended questions (see Table 4). Subsequently, the judges came to a consensus regarding which categories emerged from which questions. The three judges independently assigned a dichotomous score (0 not present; 1 present) to each theme per question.

**Table 4:** Overview of the answers of participants

Nr.	1 & 2. First reaction	3. How do you feel about the SL now?	4. Would you choose to be informed again?	5. Pregnant women should have a choice?	6. Assume child is healthy?	7. Worried about health child (1–10)
1	shocked	stigma	hesitant	yes	yes	2
2	shocked	not thinking about it often	yes	yes	yes	3
3	shocked	not thinking about it often	yes	yes	yes	3
4	worried	not thinking about it often	yes	yes	yes	2
5	shocked	not thinking about it often	yes	yes	yes	6
6	worried	not thinking about it often	yes	yes	yes	3
7^a^	shocked	not thinking about it often	yes	yes	yes	7
8	shocked	not thinking about it often	yes	yes	yes	6
9^a^	worried	not thinking about it often	hesitant	yes	yes	1
10^a^	worried	not thinking about it often	yes	yes	yes	2
11	worried	not thinking about it often	yes	yes	yes	3
12	worried	not thinking about it often	yes	yes	yes	2

^a^Still pregnant during follow-up

The observed inter-judge agreement varied between α = .44 and α = 1.00. The inter-judge reliability ranged from acceptable to excellent, except for question 1, which had a poor inter-judge reliability (α = .44) (Field 2009).

## Results: Participant’s Narratives

### Initial Experience when the SL was Disclosed

Qualitative analysis of the interviews showed that 7 out of 12 participants said they were ‘worried’, while the other 5 participants said ‘it was real shock’ to hear about the SL. We provide quotes to the answers by category. Participants marked with an asterisk (*) were still pregnant during follow-up.

#### Question 1 and 2

- What was it like for you when you were told about the SL that was found? What was your first reaction?
  - Quotes of parents who were worried (7 out of 12)
    ‘It startled me, you just don’t want to hear that about your baby. But I thought that our baby would develop normally, maybe a bit slower than others, but healthy in the end.’ (Participant 2, female)
    ‘It was unpleasant, because we thought everything would be fine. Thus far, it appears as such.’ (Participant 4, male)
    ‘We were a little shocked at first, but we understood that there was only a very small chance that something could really be wrong. So we were not too worried.’ (Participant 6, male)
    ‘We were a bit upset the first days. When we had an advanced ultrasound, everything looked normal, that was a relief to us.’ (Participant 8, male)
    ‘It came very unexpectedly, I was a little overwhelmed, but I wasn’t really, really alarmed.’ (Participant 10*, female)
    ‘At first we were not too alarmed, because the baby did not have Down syndrome. But we felt the SL diagnosis was slightly worrisome, because we did not know what we could expect at all.’ (Participant 11, male)
    ‘I was upset, because they could not tell me exactly how high the risk of developing the clinical features was. I just sat there stared at the geneticist and asked what it was, and if it was dangerous.’ (Participant 12, female)
  - Quotes of parents who were shocked (5 out of 12)
    ‘To us, it was very unclear at first. We heard something was wrong and it came as a shock, I was nervous. When we had an appointment with the geneticist to talk about it, we understood that the risk was quite low. I thought; ‘we’ll have to wait and see’, but my husband was really worried. There was a picture of a patient with the same deletion, clearly showing something was wrong. This was very upsetting to us. We didn’t really know what to do with the provided information. I wasn’t expecting it and did not think about the possibility of this kind of outcome when we engaged in prenatal diagnosis, only about the possibility of a trisomy. Maybe our older daughter has this deletion too, but she is a healthy, normal girl.’ (Participant 1, female)
    ’That was a real shock. It was quite upsetting. We thought; What is going to happen next? It was not a very nice time.’ (Participant 3, female)
    ‘We were startled, it was quite something. But we were informed of the possibility of such results.’ (Participant 5, female)
    ‘Unpleasant. It came as a shock. We did an amniocentesis hoping to hear that everything is alright, and then this susceptibility locus came as a test result. I was very emotional.’ (Participant 7*, female)
    ‘That was a real shock. It was not clear what was wrong, that made me worry a lot. The more I thought about it, the more worried I became. I had a lot of questions. I kind of panicked. Luckily, we had an appointment with the geneticist the next day. After that, I felt calmer.’ (Participant 9*, female)

#### Question 3: How do you Feel About the SL at This Very Moment?

- Quotes of parents that do not think about it often anymore (11 out of 12)
  ‘I don’t think about it too much now.’ (Participant 2, female)
  ‘I don’t think about it anymore. I think I just have a normal, healthy son.’ (Participant 3, female)
  ‘I don’t look back on it. I gave birth to a healthy son. ‘(Participant 4, female)
  ‘I like to think that nothing is wrong. At the moment, I don’t see any reason to think there is. ‘(Participant 5, male)
  ‘During pregnancy I was worried about other physical abnormalities. But now that I gave birth, I am not worried anymore.’ (Participant 5, female)
  ‘I don’t think about the SL anymore. I think I coped with the information quite well.’ (Participant 6, male)
  ‘Most abnormalities were excluded with expert ultrasound examinations. We are only unsure of other neurodevelopmental symptoms like behavioural problems. But we think everything will be fine.’ (Participant 7*, female)
  ‘After the expert ultrasound examinations we felt reassured. The SL does not have to mean anything.’ (Participant 8, male)
  ‘I think the chance of expression of the SL is very small. And if it will express itself, I think it will be mild and actionable.‘(Participant 9*, female)
  ‘We will have to wait and see. I think it is nothing very severe, since I carry it myself and do not have any symptoms. I am not too worried anymore.’ (Participant 10*, female)
  ‘I think it might be something very mild. My wife has it too. Maybe we will not even notice it. ‘(Participant 11, male)
  ‘I do not see anything out of the ordinary regarding my daughter at this point.’ (Participant 12, female)
- Quote of a parent that experienced a stigma (1 out of 12)
  ‘It is something that you keep carrying with you. If she behaves weirdly, then I immediately think that this behaviour is related to the SL. I also do not like the fact that she already had a medical file before she even was born.‘(Participant 1, female)

#### Question 4: Would you Choose to be Informed of SL Again?

- Yes (10 out of 12 parents)
  ‘I want to know as much as possible. That is the reason I chose for invasive prenatal diagnosis in the first place.’ (Participant 2, female)
  ‘Yes, even though it was distressful when we first heard about the susceptibility locus. But if something might be wrong with your child, you want to know about it.’ (Participant 4, male)
  ‘If I could choose, than I would like to know.’ (Participant 6, male)
  ‘Absolutely.’ (Participant 8, male)
  ‘Yes, I think so, because I prefer to know as much as possible. ‘(Participant 10*, female)
  ‘Personally, I want to know everything, but I have an academic degree. I can imagine that this kind of information might be very confusing for people with a lower educational level.’ (Participant 11, male)
  ‘Of course. Especially with regards to my advanced maternal age.’ (Participant 12, female)
- Hesitant (2 out of 12 parents)
  ‘If I would get pregnant again, I might not want to know. But in this pregnancy, I would not want to have missed this information.’ (Participant 1, female)
  ‘It depends if it really matters. It did give us a lot of stress, because we thought it was something very severe at first. But I would be very curious in the future (next pregnancy). A friend of mine, who had children at a young age, did not have any genetic information about her children at all. But her son has a neurodevelopmental disorder and she did not know about it in advance. It can be useful, because you know where it might come from.‘(Participant 9*, female)

Worries about the health and development of the child ranged from 2 to 7 on a 10-point scale, see Table 4. Most participants mentioned that they now ‘*just have the normal worries any parent has*’.

## Discussion

Since it has been suggested that disclosure of SL may raise emotional harm, we evaluated the psychological impact of prenatal SL disclosure on pregnant couples. Women and their partners initially felt worried and shocked. Most parents indicated that the SL was not what they had expected from invasive PND, however some recalled being informed on such possibility during the pre-test counselling. Previous research showed that pregnant women are hardly ever ready for receiving abnormal prenatal test results, even if they are well informed (Bernhardt et al. 2013; Statham et al. 2000; Lalor et al. 2009).

After their initial reaction, parents were confused and had a high need for understanding these outcomes. Most were quite alarmed by the phone call of the geneticist telling them that there was a ‘peculiar finding that needed explanation’. All parents indicated they appreciated that the post-test counselling was available the next day. Due to the highly variable penetrance and expression, the meaning of the particular finding remained uncertain for the parents. A few parents noted that this uncertainty was stressful to them at first. However, none of the parents made use of the psychological support they were offered. None of the participants felt that a termination of pregnancy was a personal option for them. The interviews revealed that some parents adopted a wait-and-see policy; that they will have to wait and see in which way their child will develop, with a positive state of mind. These parents seemed less distressed when talking about their experiences.

Parents seemed to have recovered from their initial feelings, and are now handling the knowledge about their child having a SL fairly well. They seem to base this on a seemingly normal phenotype, either after giving birth to a ‘normal appearing’ child or with the reassurance of a ‘normal’ expert fetal ultrasound examination. At the moment of the follow-up interview (mean time 10 months after disclosure), all born children had no congenital anomalies or dysmorphic features, but were still too young to be examined for neurological development. None of the fetuses had ultrasound anomalies. It is yet unknown whether these children will develop neurodevelopmental symptoms in the future. Most parents did not have lingering worries regarding the SL, except for one woman who experienced a stigma regarding her seemingly normal and healthy born daughter. She told that each time her daughter behaved aberrant, she immediately feared it might be caused by the SL. This is something that is also encountered in other studies on abnormal prenatal diagnoses, even if the child has seemingly normal appearance (Bernhardt et al. 2013). Other parents did not report stigma or enduring worries about the child’s development, and mentioned they just have ‘normal parental worries’ now. The interviewed parents indicated feeling relieved after advanced ultrasound scanning revealed no visible anomalies. We also found that parents, identified as SL carriers themselves, were in a way relieved, because they had the feeling the child could be ‘normal’ like themselves. These parents used themselves or their partner as a reference for the interpretation of the SL in their fetus. The psychological reaction reported by the individuals in our clinic may have been milder as compared to the study of Bernhardt et al. (2013). In that study, 23 participants were interviewed after disclosure of abnormal prenatal microarray results, of which 9 were known pathogenic results and 14 were variants of unknown clinical significance (VOUS). As in our study Bernhardt observed that participants initially felt shocked and worried, and had a problem with understanding the uncertainty and unquantifiable risks. Participants also shared a high need for support to manage and understand their prenatal microarray results with the help of a health care professional. However, in our study all women and their partners were counselled by a senior geneticist, whereas in the study of Bernhardt such support was not offered in all cases which might explain to the enduring concerns and a lack of support to manage decisions about termination of pregnancy and/or birth.. In both Bernhardt’s study and our own, a key factor in mitigating parental anxiety with SL disclosure appears to be post-test genetic counseling (Bernhardt et al. 2013). However, in our study, participants reactions seem milder. In the study of Bernhardt, most participants indicated that they felt their test result was ‘toxic knowledge’. In our study however, most participants indicated that they ‘just have normal parental worries now’, which clearly is a different outcome. The fact that all but one woman would choose to be informed of prenatal SL again, is a strong indicator of this.

Nearly all parents indicated they would want to be informed of SL again if offered a choice. Parents who said they preferred to know about SL, said they did so because they could quickly mobilise adequate care if needed. For instance, if developmental problems would occur, they could have access to early interventions for i.e. autism. These findings are congruent with our earlier study in which we found that a vast majority of pregnant couples, when offered a choice during pretest genetic counseling, opted for SL disclosure (van der Steen et al. 2014). The parents we discussed in this paper indicated that they would prefer to have a choice regarding the (non)disclosure of SL. This paper supports earlier reports (de Jong et al. 2013; van der Steen et al. 2014; Boormans et al. 2010) that parents highly appreciate individualized choice on the scope of prenatal testing. We have not observed psychological burden, however it has to be taken into account that the number of interviewed patients is small. Furthermore, some participants were still pregnant at the time of the interview. It is therefore difficult to make long-term conclusions. In our study, there was no distinction between prenatal de novo and inherited findings, however, due to their different nature parents might cope with them in another way. It would be interesting to evaluate this. Research on a larger scale is much needed to gain more insight in how pregnant couples are coping with this type of prenatal information.

## Conclusion

This small study showed that in our setting, there was no long-term psychological burden for pregnant couples whose fetus was diagnosed with a susceptibility locus. A key factor in mitigating parental anxiety with SL disclosure appears to be post-test genetic counseling. This paper confirms that parents highly appreciate an individualized choice on the scope of prenatal testing. We believe that if genomic microarray testing is offered, a chance of the detection of results like susceptibility loci should be routinely mentioned during pre-test counselling. An opt-out possibility may be sufficient to support the reproductive autonomy of pregnant couples.
